# Implementation of trauma-informed care and trauma-responsive services in clinical settings: a latent class regression analysis

**DOI:** 10.3389/fpsyt.2023.1214054

**Published:** 2023-10-17

**Authors:** Katherine M. Anderson, Kaitlin N. Piper, Ameeta S. Kalokhe, Jessica M. Sales

**Affiliations:** ^1^Department of Behavioral, Social, and Health Education Sciences, Emory University Rollins School of Public Health, Atlanta, GA, United States; ^2^Division of Infectious Diseases, Department of Medicine, Emory University School of Medicine, Atlanta, GA, United States; ^3^Department of Global Health, Emory University Rollins School of Public Health, Atlanta, GA, United States

**Keywords:** trauma-informed care, Ryan White HIV/AIDS clinics, HIV/AIDS, latent class analysis, implementation science

## Abstract

**Introduction:**

Engagement and retention in health care is vital to sustained health among people living with HIV (PLWH), yet clinical environments can deter health-seeking behavior, particularly for survivors of interpersonal violence. PLWH face disproportionate rates of interpersonal violence; clinical interactions can provoke a re-experiencing of the sequalae of trauma from violence, called re-traumatization. Trauma-informed care (TIC) is a strengths-based approach to case that minimizes potential triggers of re-traumatization and promotes patient empowerment, increasing acceptability of care. Yet, Ryan White HIV/AIDS clinics, at which over 50% of PLWH received care, have struggled to IMPLEMENT TIC. In this analysis, we sought to (1) identify unique sub-groups of HIV clinics based on clinical attributes (i.e., resources, leadership, culture, climate, access to knowledge about trauma-informed care) and (2) assess relationships between sub-group membership and degree of implementation of TIC and trauma-responsive services offered.

**Methods:**

A total of 317 participants from 47 Ryan White Federally-funded HIV/AIDS clinics completed a quantitative survey between December 2019 and April 2020. Questions included assessment of inner setting constructs from the Consolidated Framework for Implementation Research (CFIR), perceived level of TIC implementation, and trauma-responsive services offered by each respondent’s clinic. We employed latent class analysis to identify four sub-groups of clinics with unique inner setting profiles: Weak Inner Setting (*n* = 124, 39.1%), Siloed and Resource Scarce (*n* = 80, 25.2%), Low Communication (*n* = 49, 15.5%), and Robust Inner Robust (*n* = 64, 20.2%). We used multilevel regressions to predict degree of TIC implementation and provision of trauma-responsive services.

**Results:**

Results demonstrate that clinics can be distinctly classified by inner setting characteristics. Further, inner setting robustness is associated with a higher degree of TIC implementation, wherein classes with resources (Robust Inner Setting, Low Communication) are associated with significantly higher odds reporting early stages of implementation or active implementation compared to Weak class membership. Resourced class membership is also associated with availability of twice as many trauma-responsive services compared to Weak class membership.

**Discussion:**

Assessment of CFIR inner setting constructs may reveal modifiable implementation setting attributes key to implementing TIC and trauma-responsive services in clinical settings. Introduction.

## 1. Introduction

Engagement and retention in health care is vital to population health, yet clinical environments can reduce acceptability of care and deter health-seeking behavior. This is particularly true for individuals with experiences of interpersonal violence. Encompassing any intentional use or threat of use of power or force against another person or group that could result in injury, harm, maldevelopment, deprivation, or death (1), interpersonal violence includes, not exhaustively: sexual violence [experienced by approximately 44% of women and 23% of men in the United States (US)] (2); physical intimate partner violence (22% of US women, 14% of US men) (2); adverse childhood experienced (ACEs, 1 in 7 US children) (3); hate crimes (20% of sexual and gender minority Americans) (4); and, aggravated assault (experienced by approximately 1 in 500 people) (1). There are significant differences in exposure to violence by race, ethnicity, sexual orientation, gender identification, geographic location, and more (1).

In health care interactions, stimuli can trigger past experiences of interpersonal violence, resulting in re-traumatization or re-experiencing of the initial trauma and the physical and psychological reactions to it. Anticipation of re-traumatization can lead to health care avoidance (5), as can previous negative health care experiences (5, 6), exacerbating the negative physical and mental health outcomes associated with trauma (6–8). Providers may be unaware of their patients’ history of trauma (9), increasing the potential for unknowingly employing behaviors which may be perceived as similar to an abuser (e.g., disempowerment) (5), potentially resulting in re-traumatization. Trauma-informed care (TIC) is an evidence-based approach to care delivery that minimizes the risk of re-traumatization, thereby making care more acceptable and comfortable to patients with trauma histories (10–15), and thus may be able to improve care engagement and retention (15). Individuals with experiences of interpersonal violence who perceive greater integration of TIC in settings where they receive services endorse feeling more empowerment, having better emotional regulation, and experiencing less social withdrawal, leading to better mental and physical health outcomes (16).

People living with HIV (PLWH) experience disproportionately high rates of interpersonal violence, while also requiring frequent interaction with the healthcare system for continued antiretroviral (ART) prescription and care. Most PLWH experience intimate partner violence (IPV, 68–95% of cisgender women, 68–77% of cisgender men, 93% of transgender PLWH) (17–19), while 30% of PLWH have experienced childhood physical or sexual abuse. Additionally, PLWH experience community violence and hate crimes motivated by bias sexuality, gender identity, race, religious and social conservatism, and poverty (4, 20, 21). In addition to trauma-associated negative physical and mental health outcomes, PLWH who experience interpersonal violence have worse HIV-related outcomes, including less consistent medication adherence (22), lower CD4 cell count (22, 23), higher HIV viral load (23), and more opportunistic infections (24). Low or varied engagement in care may contribute to this, marking clinical HIV care environments as spaces in vital need of enhanced trauma-informed practices.

Despite this need, and the evidence of the positive impact of trauma-informed practices, federally-funded Ryan White HIV/AIDS clinics (RWCs) and other clinical settings have faced barriers to TIC implementation. These barriers often hinge upon characteristics of the clinical environment, including available resources such as having adequate staff and time (25, 26), leadership engagement supportive of changing practices (27, 28), availability of training on TIC (26), and networks and communications (26). The Consolidated Framework for Implementation Research (CFIR) (29) classifies these constructs as falling within the *inner setting* of the organization. Health services research demonstrates the critical nature of constructs within the inner setting for successful programmatic implementation, including of trauma-informed practices (25–28). However, most research on the inner setting characteristics of organizations focuses on the impact of individual constructs on implementation, rather than understanding the totality of inner setting attributes as they collectively contribute toward implementation of a new evidence-based practice. Improved understanding of the variable nature of the inner setting as a whole rather than individual constructs in isolation from each other can inform tailored and comprehensive combinations of strategies to promote implementation of new clinical practices, including TIC.

The goal of this study was to examine the relationship between combinations of CFIR-derived inner setting features present in RWCs and TIC implementation in these vital care settings. We employed a latent class analysis to identify subgroups of Ryan White HIV/AIDS clinics in the Southeastern US (a region with a disproportionate burden of HIV/AIDS) that have unique profiles of modifiable inner setting characteristics (based on CFIR). Identification of clinic subgroups with common inner setting strengths and gaps allows researchers to better tailor implementation strategies to the unique context of each group to strengthen delivery of TIC in these vital clinical settings serving PLWH. Therefore, the objectives of this research are (1) identify subgroups of Ryan White HIV/AIDS clinics based on their unique profiles of inner setting characteristics and (2) assess how subgroup membership is related to degree of TIC implementation and number of trauma-responsive services offered.

## 2. Materials and methods

### 2.1. Study design and setting

Data included in this analysis are from a larger mixed-methods study of CFIR inner and outer setting factors influencing the adoption and implementation of TIC in RWCs in the Southeastern US (30, 31). RWCs are federally funded clinics that provide primary care, medication, and support services to people living with HIV who are under- or uninsured; those in the Department of Health and Human Services (DHHS) Region IV (Southeastern US) serve geographic area encompassing 53% of new HIV diagnoses in the U.S. (32). These clinics vary considerably in their structural characteristics and implementation environments, spanning the states of Kentucky, Tennessee, North Carolina, South Carolina, Mississippi, Alabama, Georgia, and Florida.

### 2.2. Participants

Eligible participants were employed by an RWC in DHHS Region IV as an HIV care provider (i.e., physician, advanced practice provider), staff member (those who provide clinical and social support services, i.e., nurses, social workers, medical assistants, intake staff, and patient educators), or administrator (i.e., clinical coordinator, program manager). Participants were recruited through emails sent by regional RWC point-of-contacts, in-person at the national Ryan White providers conference, and via advertising in the newsletter for the DHHS Region IV Southeast AIDS Education and Training Center. Efforts were made to sample from all DHHS Region IV states in a representative manner through additional recruitment focused on states with a low initial participant yield. Additional details are available elsewhere (30, 31).

### 2.3. Data collection

Between December 2019 and April 2020, individuals interested in participating in the study were provided with a link to read the consent form, provide informed consent, and complete the online survey. Surveys were approximately 60 min long, self-administered, and hosted by the platform Alchemer. Participants received $30 in compensation upon completion of the survey. All study procedures were approved by the Emory University Institutional Review Board.

### 2.4. Measures

#### 2.4.1. Participant characteristics

Participants provided information about their demographic characteristics, including age, gender (female, male, non-binary), race (Black, White, other), and ethnicity (Hispanic vs. non-Hispanic). Demographic categories were collapsed due to small cell sizes. Additionally, participants were asked about their tenure at their RWC in years, and their current position: Manager/Administrator/Center Coordinator, Clinical Provider (Nurse Practitioner, Physician Assistant, certified Nurse Midwife, Physician), Nurse, Medical Assistant, Health Educator/Counselor/Patient Navigator, or Social Worker/Case Manager. Finally, personal support for TIC was operationalized using the ARTIC subscale for personal support for TIC (Cronbach *α* = 0.66), which is comprised of 5 items, each with a 7-point bipolar scale response set in which higher numbers indicate more personal support for TIC (33).

#### 2.4.2. Clinic characteristics

Participants were asked about the characteristics of their clinic, including clinic type, whether or not the clinic was academically affiliated, and whether or not mental health, substance use, and social support services were available on site. Clinic type was classified as stand-alone HIV clinic, health department, hospital-based HIV clinic, Federally Qualified Health Center, community clinic, or other. For analysis, clinic type was collapsed into hospital-based or non-hospital based. If participants from the same clinic gave disparate answers, the response with the highest frequency was applied to that clinic. Clinic state and urbanicity were determined using the US Census Bureau 2010 rural–urban classification system. Additional clinic characteristics included whether or not the following services were offered (yes/no): HIV primary care, family planning/obstetrics and gynecology (OBGYN) services, dental services, social services, substance use services, pediatric/adolescent services, and pharmacy services.

#### 2.4.3. CFIR inner setting

Implementation constructs were measured as part of a 140-item instrument adapted from the National Center of Family Homelessness’ Trauma-Informed Care Toolkit (34). Five aspects of implementation were assessed: Training and Workforce Development; Physical Environment; Screening, Assessment, and Treatment Services; Engagement and Involvement; and, Cross-sector Collaboration. Questions within each aspect were mapped onto three of the five CFIR inner setting constructs.

##### 2.4.3.1. Culture

Culture was comprised of five variables. Staff culture (14 items), patient engagement and involvement at the clinic (12 items), provider-staff communication (1 item), integration of services (1 item), and use of multidisciplinary teams (1 item) had response options on a four-point Likert scale, from “Strongly Disagree” to “Strongly Agree.” Example questions are available in Supplementary Table S1. For staff culture and patient engagement and involvement, scores from all items were summed, and then dichotomized using a median split, so that 1 = median or above and 0 = below median. A median split was chosen based on visual inspection of the distribution of data, outlined in the scoring instructions, and to account for significant skew. For provider-staff communication, integration of services, and use of multidisciplinary teams, each item was dichotomized so 1 = strongly agree and 0 = do not strongly agree, due to the distribution of the responses. For all items, respondents who indicated “I do not know” were classified as “disagree” or “below median.”

##### 2.4.3.2. Implementation climate

Implementation climate was measured using four items with response options on a five-point Likert scale, from “Strongly Disagree” to “Strongly Agree.” Example questions are available in Supplementary Table S1, and address receptivity to organizational change. Responses across the four items summed, and then dichotomized using a median split, so that 1 = median or above and 0 = below median, in line with scoring instructions and to account for skew in the distribution.

##### 2.4.3.3. Leadership engagement

Leadership engagement (3 items) was measured on a five-point Likert scale ranging from “Strongly Disagree” to “Strongly Agree.” Example questions are available in Supplementary Table S1. Responses were summed across items, and dichotomized using a median split, so that 1 = median or above and 0 = below median.

##### 2.4.3.4. Availability of resources

Availability of resources such as funding and time (5 items) was measured on a five-point Likert scale ranging from “Strongly Disagree” to “Strongly Agree.” Availability of training related to trauma (23 items) was measured on a four-point Likert scale ranging from “Strongly Disagree” to “Strongly Agree.” Example questions are available in Supplementary Table S1. For each indicator, responses across items were summed, and then dichotomized using a median split, so that 1 = median or above and 0 = below median. Ever receipt of training specifically on TIC (regardless of if the training was associated with their role(s) at the RWC) was measured using a dichotomous measure (yes/no).

##### 2.4.3.5. Knowledge of TIC

Knowledge of TIC was measured using a 10-point visual analog scale, with the prompt, “On a scale of 1–10, with 1 being no knowledge about what TIC consists of and 10 being extremely knowledgeable about what TIC consists of, how would you rate yourself?” Knowledge scores were dichotomized using a median split, so that 1 = median or above and 0 = below median.

#### 2.4.4. Implementation of TIC

Perceived degree of TIC implementation was assessed using a single ordinal item. Participants were asked, “To what extent has your clinic started to implement TIC?” Response options included “Not at all. There have been no discussions about TIC within my clinic that I’m aware of,” “We have not started yet, but we have had discussion about TIC within my clinic,” “We are in the early stages (e.g., have held a clinic-wide training, have conducted an organization assessment),” and “We are in the process of implementing new trauma-related practices or improvements (e.g., adopted enhanced trauma screening and assessment practices, adopted processes enhanced linkage to trauma-specific treatments/services).”

#### 2.4.5. Availability of trauma-responsive services

Perceived availability of trauma-responsive services was assessed using seven items. Response options were on a four-point Likert scale, from “Strongly Disagree” to “Strongly Agree.” Responses were dichotomized, so that 0 = disagree, services are not available, and 1 = agree, services are available. Respondents who indicated “I do not know” and non-responses were classified as “services are not available.” Services assessed included (1) opportunities for care coordination for services not provided within the organization; (2) education for patients about traumatic stress and triggers; (3) access to a clinician with expertise in trauma and trauma-related interventions (on-staff or available for regular consultation); (4) opportunities for patients to receive a variety of services (e.g., housing, employment, legal and educational advocacy, and health, mental health and substance abuse services); (5) referral to counseling when mental health services are needed (i.e., individual therapy, group therapy and/or family therapy); (6) opportunities for patients to express themselves in creative or nonverbal ways (i.e., art, theater, dance, movement, music); and (7) opportunities for patients to practice mindfulness, reflection, or meditation, or offers to link patients to such opportunities. Dichotomized scores for each item were summed to create a continuous measure of number of services offered, with a possible range of 0–7.

### 2.5. Statistical analysis

#### 2.5.1. Latent class analysis

Sample characteristics were computed using SAS version 9.4. Participants (*N* = 317) for whom inner setting data were available were included in the analysis. Latent class analysis, a human-centered analytic strategy that allows for identification of mutually exclusive patterns of attributes through mixture modeling, was used to identify latent classes. We ran successive fixed-effects latent class models in MPlus using Maximum Likelihood Estimation, beginning with a two-class model. All inner setting constructs detailed above were entered into the model. Successive models added one additional class each, continuing until model fit no longer improved based on the Akaike information criteria (AIC), Bayesian information criteria (BIC), sample size adjusted BIC (aBIC), entropy, and latent class probabilities. A final four-class model was selected using model fit criteria, with consideration for theoretical significance, meaningfulness of classes, and parsimony.

#### 2.5.2. Bivariate and regression analysis

Latent class membership was imported in SAS 9.4. Bivariate statistics were computed for class membership with participants characteristics and clinic structural characteristics. Using the PROC GLIMMIX function in SAS, we ran two sets of multilevel models accounting for clustering at the clinic level using a random intercept, and with covariates significant at the bivariate level (participant clinic role, dichotomized into clinical vs. non-clinical, participant race, clinic urbanicity, and clinic academic affiliation) or theoretically significant (participant personal support for TIC). First, we ran a multilevel ordinal logistic regression with the outcome variable of degree of implementation of TIC. Second, we ran a multilevel linear model with the outcome variable of number of trauma-responsive services offered.

## 3. Results

### 3.1. Latent class analysis

**Figure 1:**
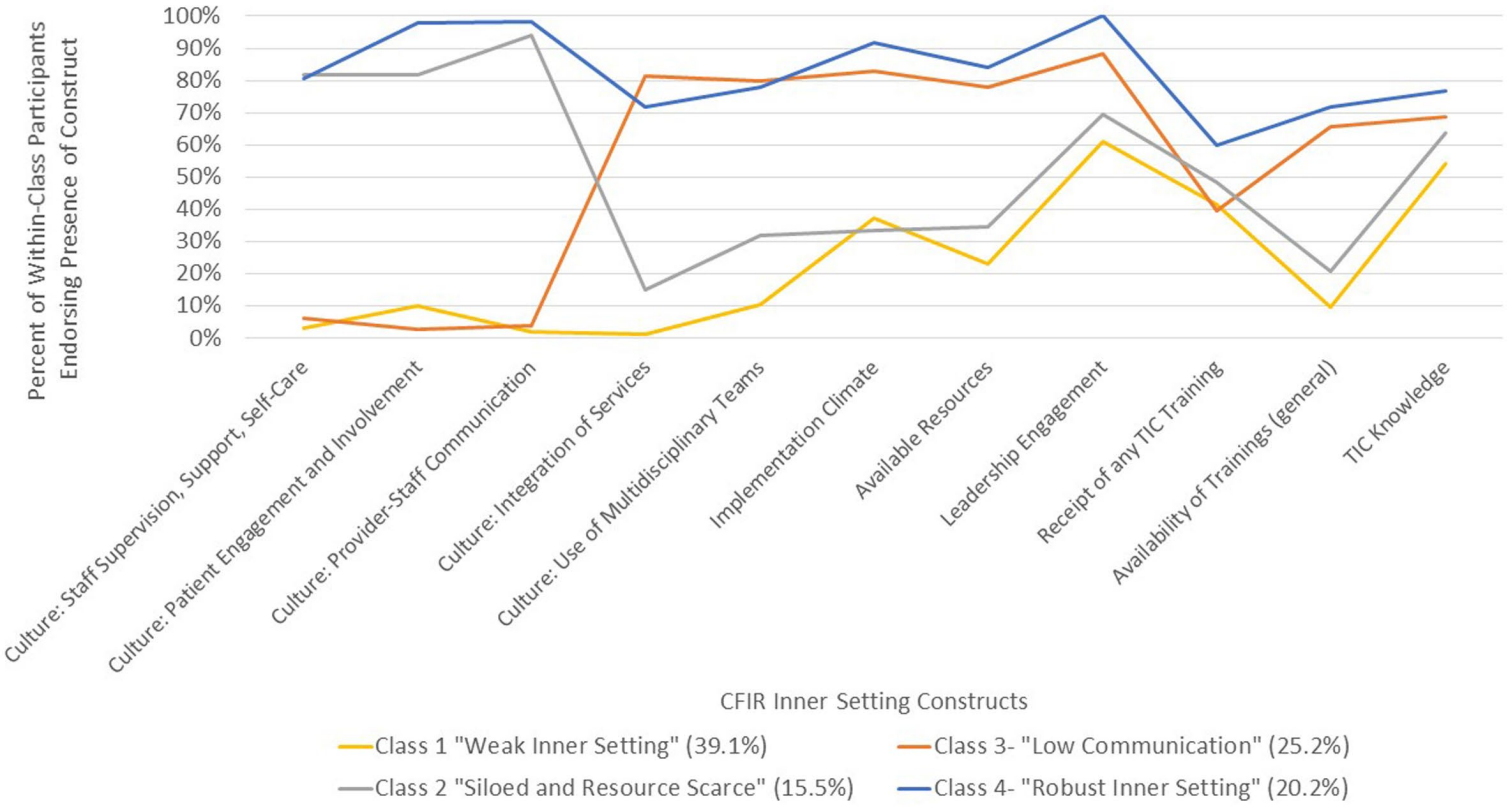
Inner setting latent variable four-class solution.

A four-class latent class model was selected as the final model, based on fit criteria (Free Parameters: 47, AIC: 3861.339, BIC: 4038.007, aBIC: 3888.934, Entropy: 0.879), latent class probabilities (0.937, 0.954, 0.905, 0.945), meaningfulness, and parsimony. The four classes demonstrated unique profiles (Figure 1). Fit statistics for all computed models are reported in Supplementary Table S2.

Class 1: Weak Inner Setting (*N* = 124) included participants whose affiliated clinics were generally low resourced, including having low access to training, and an unfavorable culture for implementation. However, approximately 60% of Class 1 participants indicated their clinical leadership was supportive of TIC implementation, and slightly over 50% had above-median knowledge of TIC. Compared to other classes, Class 1 was considered to have a “weak” inner setting.

Class 2: Siloed and Resource Scarce (*N* = 49) encompasses participants who reported a positive culture of communication, fair leadership engagement, and fair knowledge of TIC, but fewer than half reported integration of services, multidisciplinary teams, and a positive implementation climate. Further, resources, including general and TIC-specific trainings, were generally not available at these clinics.

Class 3: Low Communication (*N* = 80) included participants who rarely indicated positive culture around communication in the clinic, and only 40% of whom had received any training in TIC. However, approximately 70% had above-median knowledge of TIC, while non-communication indicators of culture, implementation climate, and other indicators were endorsed as positive by most participants.

Class 4: Robust Inner Setting (*N* = 64) contained participants who endorsed the agreement with the presence of, or above-median scores, at a rate of 60% or above for all indicators. Receipt of any training on TIC was the only indicator endorsed by less than 70% of the class. As such, Class 4 was classified as having a robust inner setting.

### 3.2. Participant characteristics

Study participants were primarily female-identifying, worked in a variety of positions in their clinic, and primarily identified as Black or White (Table 1). Participants worked at their clinic for an average of 5.66 years (SD:5.85), and were on average supportive of TIC (mean score: 5.18, possible range: 1–7). Some individual characteristics varied across latent classes- clinic roles of respondents were significantly different across classes, as were racial distribution of participants and personal support for TIC.

**Table 1:** Individual characteristics of total and by latent class membership (*N* = 317)*.

Individual characteristic	Total	Latent class				
		Class 1, “weak inner setting”	Class 2, “siloed and resource scarce”	Class 3, “low communication”	Class 4, “robust inner setting”	*p*
Admin/Office staff	44 (13.88)	12 (9.68)	4 (8.16)	20 (25.0)	9 (12.5)	**0.0065**
Clinical provider	55 (17.35)	31 (25.0)	12 (24.49)	4 (5.0)	8 (12.50)	
Health education, counselor, or patient navigator	30 (9.46)	11 (8.87)	7 (14.39)	4 (5.0)	8 (12.50)	
Manager, admin, or center coordinator	61 (19.24)	18 (14.52)	11 (22.45)	19 (23.75)	13 (20.31)	
Medical assistant/Other	38 (11.99)	14 (11.29)	4 (8.16)	13 (16.25)	7 (10.94)	
Nurse	49 (15.49)	23 (18.55)	3 (6.12)	12 (15.0)	11 (17.19)	
Social worker/Case manager	40 (12.62)	15 (12.10)	8 (16.33)	8 (10.0)	9 (14.06)	
Female (*N* = 310)	264 (85.16)	103 (85.12)	39 (81.25)	71 (89.87)	51 (82.26)	0.5084
Male	42 (13.55)	17 (14.05)	9 (18.75)	7 (8.86)	9 (14.53)	
Non-binary	4 (1.29)	1 (0.83)	0 (0.0)	1 (1.27)	2 (3.23)	
White	139 (43.85)	64 (51.61)	28 (57.14)	25 (31.25)	22 (34.38)	**0.0047**
Black	127 (40.06)	39 (31.45)	15 (30.16)	38 (47.50)	35 (54.69)	
Other	51 (16.09)	21 (16.94)	6 (12.24)	17 (21.25)	7 (10.94)	
Clinic tenure, M (SD)	5.66 (5.85)	6.06 (6.23)	5.90 (6.16)	5.377 (5.73)	5.07 (5.00)	0.6846
Personal support for TIC, *M* (*SD*) (*N* = 315)	5.18 (1.12)	4.96 (1.02)	4.80 (1.04)	5.33 (1.15)	5.72 (1.33)	**<0.0001**

*Total may not add to 100% due to missing data. Bolded values are significant at a level of *p* <0.05.

### 3.3. Associated clinic characteristics

Generally, clinics provided HIV primary care (91.8%), though infrastructure to provide other types of care was variable (Table 2). Approximately half of participants identified their clinics as being affiliated with hospital, while approximately 60% were academically affiliated, and about 65% located in urban areas. Only being affiliated with a hospital and working at a clinic in an urban context were significantly different across latent classes.

**Table 2:** Clinic characteristics by total and latent class membership (*N* = 317).

Structural characteristic		Total	Class				
			“Weak inner setting”	“Siloed and resource scarce”	“Low communication”	“Robust inner setting”	*p*-value
Academically affiliated (*N* = 277)	Yes	161 (58.12)	75 (64.66)	25 (40.48)	28 (52.54)	33 (55.00)	0.1671
	No	126 (41.88)	41 (35.34)	17 (59.52)	31 (47.46)	27 (45.00)	
HIV primary care	Yes	291 (91.80)	117 (94.35)	42 (85.71)	70 (87.50)	62 (96.88)	0.0541
	No	26 (8.20)	7 (5.65)	7 (14.29)	10 (12.50)	2 (3.13)	
Obstetrics and gynecology care	Yes	186 (58.68)	70 (56.45)	30 (61.22)	46 (57.50)	40 (62.50)	0.8457
	No	131 (41.32)	54 (43.55)	19 (38.78)	34 (42.50)	24 (37.50)	
Dental services	Yes	194 (61.20)	68 (54.84)	29 (59.18)	52 (65.00)	45 (70.31)	0.1776
	No	123 (38.80)	56 (45.16)	20 (40.82)	28 (35.00)	19 (29.69)	
Social services	Yes	233 (73.50)	91 (73.50)	38 (77.55)	53 (66.25)	51 (79.69)	0.2903
	No	84 (26.50)	33 (26.61)	11 (22.45)	27 (33.75)	13 (20.31)	
Substance use services	Yes	144 (45.43)	54 (43.55)	30 (61.22)	30 (37.50)	30 (46.88)	0.0661
	No	173 (54.57)	70 (56.45)	19 (38.78)	50 (62.50)	34 (53.13)	
Pediatric services	Yes	128 (40.38)	52 (41.94)	25 (51.02)	31 (38.75)	20 (31.25)	0.1924
	No	189 (59.62)	72 (58.06)	24 (48.98)	49 (61.25)	44 (68.75)	
Pharmacy services	Yes	228 (71.92)	87 (70.16)	39 (79.59)	55 (68.75)	47 (73.44)	0.5541
	No	89 (28.08)	37 (29.84)	10 (20.41)	25 (31.25)	17 (26.56)	
Urban	Yes	211 (66.56)	95 (76.61)	39 (79.59)	38 (47.50)	39 (60.94)	**<0.001**
	No	106 (33.44)	29 (23.39)	10 (20.41)	42 (52.50)	25 (39.06)	
Hospital affiliated	Yes	164 (51.74)	75 (60.48)	30 (61.22)	25 (31.25)	34 (53.13)	**<0.001**
	No	153 (48.26)	49 (39.52)	19 (38.78)	55 (68.75)	30 (46.88)	

Bolded values are significant at a level of *p* <0.05.

### 3.4. Implementation of TIC and trauma-responsive services

Participants identified their clinics as being approximately evenly divided between the four degrees of TIC implementation (Table 3): Not started, no discussions yet (25.87%); not started, but some discussions ongoing (22.4%); started, but in the early stages of implementation (24.61%); and started and actively implementing TIC (27.13%). Degree of implementation was statistically significantly different across classes. Participants were also asked if their clinic provided a set of trauma-responsive services in regular practice. Approximately half or more of participants agreed that each service was offered at their clinic, with clinics offering an average of 5.21 services, out of 7 services queried. Availability of each of these services, and the total number of services offered, varied significantly across latent classes.

**Table 3:** Trauma-informed care and provision of trauma services by total and latent class membership.

CFIR inner setting construct or sub-construct		Total	Class				
			“Weak inner setting”	“Siloed and resource scarce”	“Low communication”	“Robust inner setting”	*p*-value
TIC implementation	Not started, no discussions	82 (25.87)	50 (40.32)	16 (32.65)	10 (12.50)	6 (9.38)	**<0.001**
	Not started, some discussions	71 (22.40)	30 (24.19)	10 (20.41)	18 (22.50)	13 (20.31)	
	Started, early stages	78 (24.61)	29 (23.39)	13 (26.53)	21 (26.25)	15 (23.44)	
	Started, actively implementing	86 (27.13)	15 (12.10)	10 (20.41)	31 (38.75)	30 (46.88)	
Offer coordinated care for service not provided by org	Yes	278 (87.70)	94 (75.81)	44 (89.80)	79 (98.75)	61 (95.31)	**<0.001**
	No	39 (12.30)	30 (24.19)	5 (10.20)	1 (1.25)	3 (4.69)	
Offer education about trauma	Yes	189 (59.62)	37 (29.84)	25 (51.02)	70 (87.06)	57 (89.06)	**<0.001**
	No	128 (40.38)	87 (70.16)	24 (48.98)	10 (12.50)	7 (10.94)	
Offer access to clinician with trauma expertise	Yes	235 (74.13)	65 (52.42)	36 (73.47)	72 (90.00)	62 (96.88)	**<0.001**
	No	82 (25.87)	59 (47.58)	13 (26.53)	8 (10.00)	2 (3.13)	
Offer access to a variety of services	Yes	283 (89.27)	97 (78.23)	45 (91.84)	78 (97.50)	63 (98.44)	**<0.001**
	No	34 (10.73)	27 (21.77)	4 (8.16)	2 (2.50)	1 (1.56)	
Offer referrals to mental health services	Yes	300 (94.64)	108 (87.10)	48 (97.96)	80 (100.0)	64 (100.0)	**<0.001**
	No	17 (5.36)	16 (12.90)	1 (2.04)	0 (0.0)	0 (0.0)	
Offer opportunities for creative expression	Yes	158 (49.84)	37 (29.84)	15 (30.61)	63 (78.75)	43 (67.19)	**<0.001**
	No	159 (49.84)	87 (70.16)	34 (69.39)	17 (21.25)	21 (32.81)	
Offer opportunities for mindfulness	Yes	209 (65.93)	56 (45.16)	26 (53.06)	70 (87.50)	57 (89.06)	**<0.001**
	No	108 (34.07)	68 (54.84)	23 (46.94)	10 (12.50)	7 (10.94)	
Average # services offered	N (SD)	5.21 (1.91)	3.98 (1.98)	4.88 (1.56)	6.40 (1.43)	6.36 (0.95)	**<0.001**

Bolded values are significant at a level of *p* <0.05.

Table 4 presents the results of multilevel ordinal logistic regressions with level of implementation of TIC as the dependent variable. Compared to participants in Low Communication (class 3) clinics, Weak Inner Setting (class 1) clinic participants had 3.6 times the odds of judging their clinic as not yet actively implementing TIC, 4.4 times the odds of not yet being in the early stages of implementing TIC, and 4.8 times the odds of not yet having discussions about TIC (aOR = 3.677, 95% CI:2.017, 6.703; aOR = 1.366, 95% CI:2.16, 8.60; aOR = 4.788, 95% CI:2.061, 11.122). These differences were even more stark compared to participants in clinics with Robust Inner Setting (class 4) clinics, wherein Weak Inner Setting (class 1) clinic participants had 4.5 to 6 times higher odds of reporting not yet actively implementing TIC, not yet being in the early stages of TIC implementation, or not yet having discussions about TIC (aOR = 4.560 95% CI:1.379, 8.741; aOR = 4.912 95% CI:2.281, 10.576; aOR = 6.044, 95% CI:2.164, 16.879, respectively). Similar, though slightly lower magnitude associations were demonstrated when comparing Siloed and Resource Scare (class 2) clinics to Low Communication (class 3) clinic participants (aOR = 2.192, 95% CI:1.070, 4.489; aOR = 2.474, 95% CI:1.116, 5.480; aOR = 3.661, 95% CI:1.366, 9.812) and Robust Inner Setting (class 4) clinic participants (aOR = 2.718, 95% CI:1.262, 5.856; aOR = 2.783, 95% CI:1.148, 6.745; aOR = 4.622, 95% CI:1.459, 14.640). There were no statistically significant differences in the odds of implementation level between Inner Setting Weak (class 1) and Siloed and Resource Scarce (class 2) clinics, nor were there between Low Communication (class 3) and Robust Inner Setting (class 4) clinics.

**Table 4:** Odd ratios of implementation stage threshold by latent class membership.

	Odds of implementation stage below “actively implementing”		Odds of implementation stage below “early stages”		Odds of implementation stage below “some discussions”	
Inner setting class	aOR	95% CI	aOR	95% CI	aOR	95% CI
1 vs. 2 (ref)	1.678	0.875, 3.216	1.765	0.871, 3.576	1.308	0.598, 2.862
1 vs. 3 (ref)	**3.677**	**2.017, 6.703**	**4.366**	**2.216, 8.601**	**4.788**	**2.061, 11.122**
1 vs. 4 (ref)	**4.560**	**2.379, 8.741**	**4.912**	**2.281, 10.576**	**6.044**	**2.164, 16.879**
2 vs. 3 (ref)	**2.192**	**1.070, 4.489**	**2.474**	**1.116, 5.480**	**3.661**	**1.366, 9.812**
2 vs. 4 (ref)	**2.718**	**1.262, 5.856**	**2.783**	**1.148, 6.745**	**4.622**	**1.459, 14.640**
3 vs. 4 (ref)	1.240	0.640, 2.405	1.125	0.504, 2.513	1.262	0.402, 3.962

Bolded values are significant at a level of *p* <0.05.

Compared to participants in Weak Inner Setting (class 1) clinics, participants in Low Communication (class 3) and Robust Inner Setting (class 4) clinics reported significantly higher number of services available (Table 5), with an average of 2.24 additional services available in Low Communication inner setting clinics (95% CI: 1.7825, 2.6988) and an average of 2.17 additional services available in Robust Inner Setting clinics (95% CI: 1.6781, 2.6607).

**Table 5:** Beta estimates for number of trauma-responsive-services by latent class membership.

Inner setting type	Beta	95% CI
Class 1	Ref	**–**
Class 2	0.8166	0.3063, 1.3268
Class 3	**2.2406**	**1.7825, 2.6988**
Class 4	**2.1694**	**1.6781, 2.6607**

Bolded values are significant at a level of *p* <0.05.

## 4. Discussion

The Ryan White network is a robust system of federally-funded clinics offering HIV clinical and support services across the US. They are subject to centralized training, evaluation, and feedback mechanisms, which enable systematic implementation of new programs, including TIC. A key part of ending the HIV epidemics, RWCs serve the plurality of PLWH in the US, warranting support in their efforts. While regulations within the Ryan White System may produce a level of similarity across RWCs, these clinics are located across a variety of geographic, demographic, political, and workforce contexts, which significantly influence the clinical inner setting. Using latent class analysis, we identified four sub-groups of RWCs based on CFIR inner setting constructs, and assessed their associations with degree of implementation of TIC and availability of trauma-responsive services. One group endorsed limited inner setting strength (“Weak Inner Setting”), which was associated with being the least likely to have begun implementing TIC, and offering the fewest trauma responsive services. Two groups (“Low Communication” and “Siloed and Resource Scarce”) demonstrate moderate inner setting strength, with variability in which inner setting constructs were identified by participants; “Low Communication” inner setting clinics had higher odds of actively implementing TIC than “Weak Inner Setting” clinics and offered more trauma-responsive services, while “Siloed and Resource Scarce” settings did not. Membership in the “Robust Inner Setting” group was associated with the highest odds of implementation. Tailoring implementation strategies to address the needs of these sub-group will facilitate more effective implementation of TIC in RWCs.

Approximately 20% of clinics are classified within the “Robust Inner Setting” sub-group, approximately 70% of which are already implementing TIC (early stages or actively implementing). These clinics likely need the least support in beginning or continuing TIC. Training and technical assistance may be sufficient to enhance their implementation, with a focus on TIC-specific training and knowledge generation, which were endorsed as the weakest constructs for this group.

“Low Communication” RWCs- classified as having moderate inner setting strength- endorsed slightly lower levels of active implementation than strong inner setting clinics (39% vs. 47%), but similar numbers of trauma-responsive services. “Low Communication” clinics were more likely to be located in rural areas, and more likely to not be affiliated with a hospital than other clinics; the lack of communication infrastructure demonstrated by “Low Communication” clinics may be related to relative the isolation of these clinics, while other clinics benefit from hospital systemic-integrated communication pathways. Representing approximately 25% of the sample, “Low Communication” clinics endorsed similar availability of training and other resources, leadership engagement, implementation climate, integration of services, and use of multidisciplinary teams, yet a distinguishing difference was they reported markedly lower staff supportive practices such as trauma-informed supervision, support, and self-care practices, patient engagement and involvement, and provider-staff communication in comparison to strong inner setting clinics. This may indicate that while resources, leadership engagement, and service integration are sufficient for establishing and/or maintaining availability of trauma-responsive services, such as connecting patients to onsite mental or behavioral health services, strategies to enhance communication between providers, staff, and patients may be necessary to facilitate robust implementation of patient-centered trauma screening, assessment and coordinated care. Critically, evidence suggests that working with patients with trauma and complex needs can contribute to compassion fatigue, secondary stress and burnout (35–38) and provider burnout has been associated with lower willingness to learn new skills and implement organizational change (39–41). Thus, “Low Communication” clinics may benefit from support in developing robust staff support systems, including opportunities for interpersonal and professional communication such as support groups and clinic-wide meetings that address stress, compassion fatigue and burnout. Also, to ensure new practices for screening, assessing and addressing trauma in clinical care are acceptable to patients and not unintentionally retraumatizing, these clinics should identify methods for patient feedback, such as evaluation surveys or community advisory boards.

“Siloed and Resource Scarce” clinics, encompassing approximately 15% of the sample, were also considered to have a moderate implementation climate, with endorsement of a positive culture of communication, but had fewer multidisciplinary teams, integration of services, and lower availability of resources, including trainings, than “Robust Inner Setting” or “Low Communication” sub-groups. Implementation of TIC in these clinics had progressed further than in the “Weak Inner Setting” groups, but less than in clinics with greater resource availability (including “Low Communication” and “Robust Inner Setting” groups), indicating that resource availability may act as a threshold that must be met for progression along the TIC implementation continuum. In addition to training and technical assistance, leaders in “Siloed and Resource Scarce” clinics may need guidance or support from an external facilitator on how to integrate TIC practices within the constraints of their clinic and without overburdening staff, such as using a referral model for providing TIC services. Further, without many onsite services, focusing on adopting a referral model for providing TIC services may be ideal, which would require the building or expanding of external partnerships.

“Weak Inner Setting” clinics, by contrast, had low endorsement of most inner setting constructs, though over 50% indicated above-median leadership engagement and knowledge of TIC. Most clinics within this sub-group (approximately 65%) reported their clinics had conversations about TIC implementation, but had not progressed to any level of implementation. These clinics would likely require multiple strategies to overcome barriers to adopting and implementing TIC, requiring a more comprehensive implementation plan than other clinics. Notably, approximately 40% of RWCs surveyed were classified as “Weak Inner Setting,” underscoring the high need for implementation support and resources in RWCs in the Southern U.S.

Despite differences across sub-groups, most clinics were providing at least some trauma-responsive services, such as offering coordinated care for services not provided by their organization (87.7%), offering referrals to mental health services (94.6%), and offering access to a variety of services beyond HIV care (89.3%). This is congruent with the Ryan White model of care, in which integrated service delivery is inherent to clinical structure. However, beyond the infrastructure provided by the RW network for base integrated services, variability in service offering is observable. Clinics with moderate “Low Communication” settings had higher odds of implementing TIC than “Weak Inner Setting” clinics, while “Siloed and Resources Scarce” clinics did not. This suggests that strengthening inner setting constructs in general facilitated TIC implementation, though some may be more effective than others.

There are limitations to the current study, including the use of non-probability sampling and self-administration of the survey, which may have resulted in incomplete or biased responses. Dichotomization of indicator variables reduces variability in responses that may be important for a more nuanced understanding of inner setting factors influential for implementation. Specifically, this could lead to a conservative estimate of the influence of these factors; further research should employ innovative methods for better understanding these latent variables. Similarly, the use of mode to categorize level of implementation of TIC may limit the nuance in assessment of implementation, but provided what could be considered closest to a consensus measure of the standard measures of central tendency. The reliability of the ARTIC assessment of personal support for TIC was lower than desirable, which may indicate heterogeneity of the items, or that the items do not form a single construct; however, the measures has been previously validated and all items were retained for comparison with extant literature. Additionally, all “do not know” responses were classified as below median, to minimize assumptions about presence of constructs. This may lead to underestimation of the salience of these constructs in certain settings, exaggerating the importance of factors not reported as “do not know.” However, lack of awareness of constructs by clinic members may indicate insufficient salience or diffusion of the constructs, particularly when inner setting constructs involve all individuals in the clinic setting and are likely to be perceived by staff and providers. To facilitate interpretation of results, large numbers of indicators variable are not recommended for use in latent class analysis; therefore, this analysis does not include factors other than the inner setting, such as the environment in which RWCs exist, qualities of the intervention itself, the process of implementation, or characteristics of individuals implementing or receiving TIC. These factors may be highly influential in implementation, and may have interactive effects with the inner setting. However, research has consistently identified the importance of clinical inner settings for implementation (28–30), and latent class analysis allows for identification of latent patterns of inner setting characteristics that are the most salient, facilitating identification of modifiable targets for implementation technical assistance.

In conclusion, despite many common elements across RWCs stemming from requirements from the Ryan White HIV/AIDS program, there is discernable variation in the internal operating characteristics of RWCs across the Southeastern US which is associated with TIC implementation within this vital safety-net clinical network. Identifying sub-groups of RWCs with similar internal strengths and weakness pertaining to TIC implementation can facilitate tailoring of strategies to each subgroups’ needs to facilitate and strengthen TIC implementation. To enable the transition to trauma-informed HIV care across the Ryan White clinical network, the Health Resources and Services Administration (HRSA) and other training and oversight agencies for the Ryan White HIV/AIDS program could offer tailored packages of strategies to RWCs thereby giving them the right tools they need to enhance internal operations for TIC implementation in their clinic. Future studies should build upon the completed objectives of this research, and evaluate the utility of these tailored strategies on TIC adoption and implementation across RWCs.

## Data availability statement

The raw data supporting the conclusions of this article will be made available by the authors, upon reasonable request.

## Ethics statement

The studies involving humans were approved by the Emory University Institutional Review Board. The studies were conducted in accordance with the local legislation and institutional requirements. The participants provided their written informed consent to participate in this study.

## Author contributions

KA: methodology, formal analysis, writing- original draft, writing- reviewing and editing. KP: formal analysis, writing-original draft, writing- review and editing. AK: methodology, writing-review and editing, supervision, funding acquisition. JS: conceptualization, methodology, investigation, resources, writing- original draft, writing- review and editing, supervision, funding acquisition. All authors contributed to the article and approved the submitted version.
