# Supplementary material for: Implementation of trauma-informed care and trauma-responsive services in clinical settings: a latent class regression analysis
Source: Front Psychiatry. 2023 Oct 17;14:1214054. doi: 10.3389/fpsyt.2023.1214054 (PMC10616460; doi:10.3389/fpsyt.2023.1214054)
Supplement: Supplementary file 1 [file Table_1.DOCX]

| Supplemental Table 1. CFIR Inner Setting Construct Measurement: Questions | |
| --- | --- |
| Construct | Example Questions |
| Culture: Staff Culture | **Prompt:** Read each item and rate it from “strongly disagree” to “strongly agree,” using your initial impression. **Remember you are evaluating your RWPCC not your individual performance.**   1. Staff members have regular team meetings. 2. Topics related to trauma are addressed in team meetings. 3. Topics related to self-care are addressed in team meetings (i.e. vicarious trauma, burn-out, stress-reducing strategies). 4. Self-care is encouraged and supported with policy and practice at your RWPCC. 5. Staff members meet with their supervisor/director regularly. 6. Staff members receive individual supervision from someone who is trained in understanding trauma. 7. Part of staff’s time with their supervisor/director is used to help staff members understand their own stress reactions. 8. Part of the staff’s time with their supervisor/director is used to help staff members understand how their stress reactions impact their work with patients. 9. Your RWPCC helps staff members debrief after a crisis. 10. Staff have adequate support in dealing with challenging client situations. 11. Supervisors have an understanding of the emotional impact (burnout, vicarious trauma, and compassion fatigue) associated with their work. 12. Your RWPCC provides opportunities for staff input into program practices. 13. The actions that follow (solicitation of input) demonstrate that staff have been heard. 14. Supervisors communicate that staff members’ opinions are valued even if they are not always implemented. |
| Culture: Patient Engagement | **Prompt:** Read each item and rate it from “strongly disagree” to “strongly agree,” using your initial impression. **Remember you are evaluating your RWPCC not your individual performance.**   1. The organization reviews rules, rights, and grievance procedures with patients regularly. 2. Patients are informed about how your RWPCC responds to personal crises (i.e. suicidal statements, violent behavior and mandatory reports). 3. Patients’ rights are posted in places that are visible (i.e. room checks, grievance policies, mandatory reporting rules). 4. Materials are posted about traumatic stress (i.e. what it is, how it impacts people, and available trauma-specifics resources). 5. Your RWPCC has regularly scheduled procedures and opportunities for patients to provide input. 6. Your RWPCC has effective policies in place to handle any changes in schedules. 7. Your RWPCC is flexible with procedures if needed, based on individual patient circumstances. 8. Patients are given opportunities to evaluate your RWPCC and offer their suggestions for improvement in anonymous and/or confidential ways (i.e. suggestion boxes, regular satisfaction surveys, meetings focused on necessary improvements, etc.) 9. Your RWPCC recruits patients to serve in an advisory capacity. 10. Patients are invited to share their thoughts, ideas and experiences with your RWPCC. 11. Patients have opportunities to become involved in the development of your RWPCC activities. 12. Patients are involved in providing services (i.e. peer-run support groups, educational, and therapeutic groups.) |
| Culture: Provider-Staff Communication | **Prompt:** Read each item and rate it from “strongly disagree” to “strongly agree,” using your initial impression. **Remember you are evaluating your RWPCC not your individual performance.**   1. Policies and procedures encourage providers and staff to have regular contact (with consent of the patient) with other care providers who serve the same patient. |
| Culture: Integration of Services | **Prompt:** Read each item and rate it from “strongly disagree” to “strongly agree,” using your initial impression. **Remember you are evaluating your RWPCC not your individual performance.**   1. Care management that integrates substance abuse, mental health, and violence/trauma services is available. |
| Culture: Multidisciplinary Teams | **Prompt:** Read each item and rate it from “strongly disagree” to “strongly agree,” using your initial impression. **Remember you are evaluating your RWPCC not your individual performance.**   1. Multi-disciplinary teams can be consulted to address service plan difficulties. |
| Implementation Climate | **Prompt:** Read each item and rate it from “strongly disagree” to “strongly agree,” using your initial impression. **Remember you are evaluating your RWPCC not your individual performance.** **Individuals (i.e. providers, administrators, and staff) in my clinic:**   1. Have a sense of personal responsibility for improving patient care and outcomes 2. Cooperate to maintain and improve effectiveness of patient care 3. Are willing to innovate and/or experiment to improve clinical procedures 4. Are receptive to change in clinical processes |
| Leadership Engagement | **Prompt:** Read each item and rate it from “strongly disagree” to “strongly agree,” using your initial impression. **Remember you are evaluating your RWPCC not your individual performance. Senior leadership/clinical management in my clinic:**   1. Reward clinical innovation and creativity to improve patient care 2. Solicit opinions of clinical staff regarding decisions about patient care 3. Seek ways to improve patient education and increase patient participation in treatment |
| Availability of Resources | **Prompt:** Read each item and rate it from “strongly disagree” to “strongly agree,” using your initial impression. **Remember you are evaluating your RWPCC not your individual performance.** **In my clinic, when there is agreement that change needs to happen:**   1. We have the necessary support in terms of budget or financial resources. 2. We have the necessary support in terms of training. 3. We have the necessary support in terms of facilities. 4. We have the necessary support in terms of staffing. |
