# Supplementary material for: Implementation of trauma-informed care and trauma-responsive services in clinical settings: a latent class regression analysis
Source: Front Psychiatry. 2023 Oct 17;14:1214054. doi: 10.3389/fpsyt.2023.1214054 (PMC10616460; doi:10.3389/fpsyt.2023.1214054)
Supplement: Supplementary file 2 [file Table_2.DOCX]

| Supplemental Table 2. Ryan White Clinic Inner Setting Factor Latent Class Analysis Fit Statistics | | | | | |
| --- | --- | --- | --- | --- | --- |
|  | 2-Class Model | 3-Class Model | 4-Class Model | 5-Class Model | 6-Class Model |
| Number of Free Parameters | 23 | 35 | 47 | 59 | 71 |
| AIC | 4151.062 | 3917.175 | 3861.339 | 3845.325 | 3838.120 |
| BIC | 4237.517 | 4048.736 | 4038.007 | 4067.100 | 4105.002 |
| aBIC | 4164.566 | 3937.725 | 3888.934 | 3879.966 | 3879.806 |
| Entropy | 0.938 | 0.893 | 0.879 | 0.853 | 0.871 |
| Average Latent Class Probabilities for Most Likely Latent Class Membership | 0.974; 0.992 | 0.976; 0.966; 0.933 | 0.937; 0.954; 0.904; 0.945 | 0.901; 0.959; 0.857; 0.856; 0.954 | 0.932; 0.931; 0.870; 0.914; 0.965  0.898 |
